# ESI–MS analysis of Cu(I) binding to apo and Zn_7_ human metallothionein 1A, 2, and 3 identifies the formation of a similar series of metallated species with no individual isoform optimization for Cu(I)

**DOI:** 10.1093/mtomcs/mfae015

**Published:** 2024-03-19

**Authors:** Adyn Melenbacher, Martin J Stillman

**Affiliations:** Department of Chemistry, The University of Western Ontario, London, Ontario N6A 5B7, Canada; Department of Chemistry, The University of Western Ontario, London, Ontario N6A 5B7, Canada

**Keywords:** Cu homeostasis, Cu-thiolate clusters, ESI–MS, metallothionein, phosphorescence, Zn homeostasis

## Abstract

Metallothioneins (MTs) are cysteine-rich proteins involved in metal homeostasis, heavy metal detoxification, and protection against oxidative stress. Whether the four mammalian MT isoforms exhibit different metal binding properties is not clear. In this paper, the Cu(I) binding properties of the apo MT1A, apo MT2, and apo MT3 are compared and the relative Cu(I) binding affinities are reported. In all three isoforms, Cu_4_, Cu_6_, and Cu_10_ species form cooperatively, and MT1A and MT2 also form a Cu_13_ species. The Cu(I) binding properties of Zn_7_-MT1A, Zn_7_-MT2, and Zn_7_-MT3 are compared systematically using isotopically pure ^63^Cu(I) and ^68^Zn(II). The species formed in each MT isoform were detected through electrospray ionization–mass spectrometry and further characterized using room temperature phosphorescence spectroscopy. The mixed metal Cu, Zn species forming in MT1A, MT2, and MT3 have similar stoichiometries and their emission spectral properties indicate that analogous clusters form in the three isoforms. Three parallel metallation pathways have been proposed through analysis of the detailed Cu, Zn speciation in MT1A, MT2, and MT3. Pathway ① results in Cu_5_Zn_5_-MT and Cu_9_Zn_3_-MT. Pathway ② involves Cu_6_Zn_4_-MT and Cu_10_Zn_2_-MT. Pathway ③ includes Cu_8_Zn_4_-MT. Speciation analysis indicates that Pathway ② is the preferred pathway for MT2. This is also evident in the phosphorescence spectra with the 750 nm emission from Cu_6_Zn_4_-MT being most prominent in MT2. We see no evidence for different MT isoforms being optimized or exhibiting preferences for certain metals. We discuss the probable stoichiometry for MTs *in vivo* based on the *in vitro* determined binding constants.

## Graphical Abstract

**Graphical Abstract:**
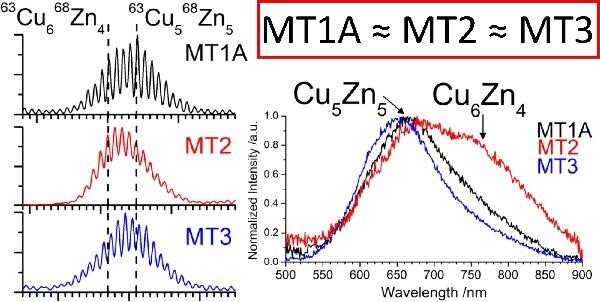
Species with the same stoichiometry and similar emission wavelengths form in MT1A, MT2, and MT3.

## Introduction

**Fig. 1:**
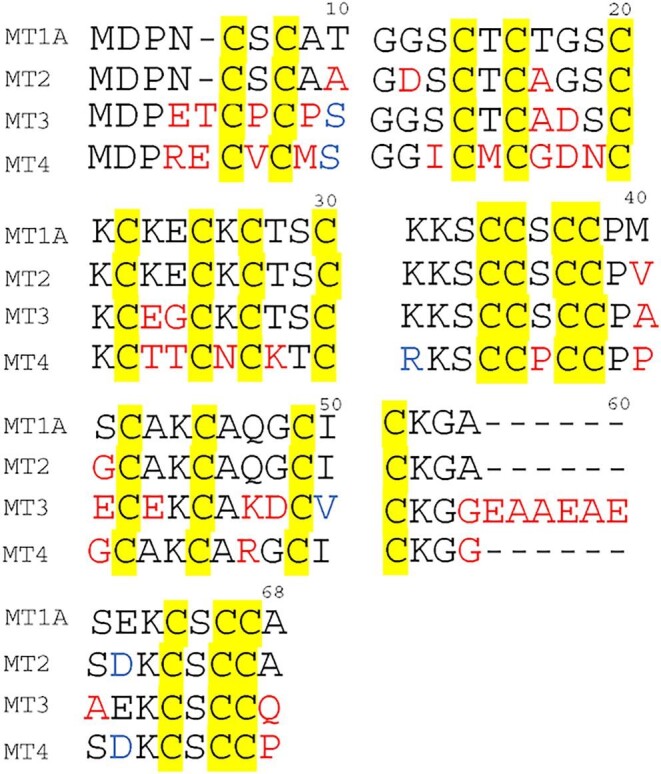
Comparison of human MT1A (Uniprot #**P04731**), MT2 (Uniprot #**P02795**), MT3 (Uniprot #**P25713**), and MT4 (sequence from Moleirinho *et al.*^2^) amino acid sequences. Amino acids labeled in red are different compared to the corresponding amino acid in MT1A. Amino acids labeled in blue are similar amino acids to the corresponding amino acid in MT1A.

Metallothionein (MT) proteins are cysteine-rich proteins involved in Cu(I) and Zn(II) homeostasis, heavy metal detoxification, and protection against oxidative stress. The importance of MTs is emphasized by their presence across multiple domains of life. In mammals, there are four MT isoforms, MT1, MT2, MT3, and MT4 (Fig. 1). All mammalian MTs contain 20 cysteines. These cysteines are divided into a β domain containing 9 cysteines and an α domain containing 11 cysteines based on the two domain structures that form when seven divalent metals bind to mammalian MTs.^1^

The multiple MT isoforms originate from gene duplication events prior to the evolution of mammals. The first gene duplication event resulted in the divergence of MT4 from the ancestor of MT1, MT2, and MT3. A second duplication event then separated the ancestor of MT1 and MT2 from MT3. Interestingly, while there is one copy of the MT2, MT3, and MT4 genes, there are 13 copies of the MT1 gene in the human genome, where each copy is considered a sub isoform of MT1. Five of these duplicate copies are pseudogenes meaning that they do not encode a functional protein resulting in eight functional sub isoforms of MT1.^2^ The amino acid sequence of MT3 differs significantly compared to the other MT isoforms. It has a threonine insert at position 5 and a hexapeptide insert in the α domain. The CPCP motif (residues 6-9), specific to MT3, results in an additional growth inhibitory function in neurons.^3^ MT4 also has an additional glutamic acid in the β domain compared to MT1 and MT2. MT4 is suggested to play a role in zinc metabolism during differentiation of stratified epithelial cells.^4^

In humans, MT1 and MT2 are expressed throughout the body.^5^ MT2 exhibits higher basal expression than the MT1 sub isoforms, which have different expression patterns depending on the sub isoform.^5^ MT3 is primarily found in the central nervous system,^6^ but has been found to be expressed in other locations as well.^7,8^ MT4 expression seems to be the most limited where it is expressed strictly in epithelial cells.^4^

The presence of multiple MT isoforms and their different expression patterns leads directly to the question of whether these isoforms have different functions and/or metal binding properties. Under normal physiological conditions, MTs tend to be isolated with Zn(II) and Cu(I).^6,9–15^ Upon exposure to Cd(II), MTs in the liver and kidneys can accumulate Cd(II).^11,16,17^ Early research on MT1 and MT2 found that MT1 and MT2 expression responded differently to glucocorticoids, Zn(II), as well as Cd(II). While MT2 was found to be highly inducible by glucocorticoids and Zn(II), MT1A was found to only be highly stimulated by the presence of Cd(II). This led to the hypothesis that MT1 and MT2 have different functions with MT1 serving a protective role and MT2 being involved in the homeostasis of Cu(I) and Zn(II).^18^ These potentially different functions may arise from differences in the expression or, as some have suggested, differences in the metal binding properties of the two MT isoforms.^19^ MT2 is less susceptible to proteolytic breakdown compared to MT1.^20^ This may also be due to differences in the metallation properties, as metal-thiolate clusters in MT can impede proteolysis.^21^ MT2 has been shown to bind Zn(II) and Cd(II) ions with higher affinity compared to MT3.^22^

Differences in the metal binding character have been reported for mouse MT1, MT2, and MT3 and these isoforms have been described as having either “Cu-thionein character” or “Zn-thionein character.”^19,23^ In these studies, the metal binding properties were analysed by expressing the recombinant MT proteins in *Escherichia coli* (*E. coli*) in the presence of either Zn(II), Cd(II), or Cu(I) and the resulting products were isolated and purified before electrospray ionization (ESI)–mass spectral analysis.^19,23^ While there were no issues isolating Cd_7_-mouse MT2 and Zn_7_-mouse MT2 from the *E. coli*, the researchers had difficulty isolating Cu-mouse MT2 and concluded that mouse MT2 had a “poor performance for Cu(I) coordination” unlike mouse MT1.^19^ Our recent results, however, show no issues binding Cu(I) to Zn_7_ human MT2^24^ or rabbit liver MT2^25^  *in vitro* (and in this paper we demonstrate that there are no issues with Cu(I) binding to apo MT2). It is possible that the poor Cu(I) binding reported previously^19^ was due to difficulties in isolating the very air sensitive products. On the other hand, mouse MT3 was reported to be more suited to Cu(I) binding than MT1 and MT2 by the same method.^23^ However, it is difficult to draw conclusions about the metal binding properties from the methods used due to additional possible complications from variations in metal concentrations within the cells as well as the possibility for disruption of the metallation status upon purification, e.g. through protein oxidation. While *in vitro* Cu(I) replacement studies of Zn_7_-MTs were carried out,^19,23^ the authors used pH changes to distinguish Cu(I) and Zn(II) binding in the ESI–mass spectra, which would also lead to metal rearrangement as the structures adopted by Cu(I) have been shown to be very pH dependent.^19,23,26^

We have recently reported on the exact Cu, Zn ratios that result upon addition of ^63^Cu(I) to ^68^Zn_7_-MT1A,^27^  ^68^Zn_7_-MT2,^24^ and ^68^Zn_7_-MT3^28^ and so in our present report we are able to, for the first time, compare the metallation properties of the three isoforms in much greater detail. We note that more extensive summaries of the previous studies of Cu(I) binding to MT1A, MT2, and MT3 were included in the three original publications^24,27,28^ and that a detailed historical record is not repeated here although we do include a commentary on the issues inherent in some of the previous reports with respect to determining exact Cu: Zn ratios bound.

In this paper, we present a comprehensive comparison of the Cu, Zn binding properties of MT1A, MT2, and MT3 through analysis of the species formed after the addition of ^63^Cu(I) to the ^68^Zn_7_-MT1A, ^68^Zn_7_-MT2, and ^68^Zn_7_-MT3 as well as the addition of naturally abundant Cu(I) to apo MT1A, MT2, and MT3. We have included novel Cu(I) binding data to apo MT2 to complete the full set of Cu(I) binding data for each of the three major human isoforms. From the array of detailed speciation data for these three prominent human MT isoforms, we can identify recurring species that form starting from the novel Cu_1_Zn_7_-MT1A/2/3. Analysis of the subsequent, cooperatively formed species leads to the proposal that the same two or three metallation pathways are active in each isoform.

## Methods

### Recombinant protein expression and purification

Cd-saturated MT1A, MT2, and MT3 were produced recombinantly using *E. coli* and purified according to the procedures published in Melenbacher *et al.*^27^ and Melenbacher and Stillman,^24,28^ respectively.

### MT metallation

All ammonium formate solutions were saturated with argon and contained 0.1 mM tris(2-carboxyethyl)phosphine (TCEP) (Soltec Ventures, Beverly, MA, USA) to prevent oxidation of the thiols. The cadmium was removed from the MT proteins to form the apo MT using a PD10 column (Cytiva) equilibrated to pH 2 using 10 mM pH 2 ammonium formate. The released Cd(II) was separated from the MT through buffer exchange carried out by centrifuging the protein at 4000 ×*g* using a 3 kDa centrifugal filter (Amicon, Burlington, MA, USA). The pH was increased using dilute argon-saturated NH_4_OH and a second PD10 column was used to bring the pH to neutral pH. While this method completely removes the original cadmium, slight zinc contamination can be observed by the very sensitive electrospray ionization–mass spectrometry (ESI–MS). This is due to the MT scavenging the unavoidable trace contamination of zinc despite acid washing of glassware. In between each step, the protein was also thoroughly evacuated and argon-saturated to avoid oxidation. The concentration of the apo protein was measured by metallating a fraction of the protein sample with cadmium and measuring the absorbance at 250 nm (S-Cd ligand to metal charge transfer band) using a molar absorption coefficient of 89 000 M^−^^1 ^cm^−1^.

^68^ZnO was purchased from Trace Sciences International (Richmond Hill, Ontario, Canada) and dissolved in dilute acetic acid (Caledon Laboratory Chemicals, Georgetown, Ontario, Canada) at approximately 60°C. Dilute NH_4_OH was used to increase the pH to 4.1 and the ^68^Zn(II) was diluted to a final concentration of 10 mM using Milli Q water. A total of 7.5 mol. eq. of argon-saturated ^68^Zn(II) was added to the apo MT to ensure all of the protein was saturated with ^68^Zn(II) to form ^68^Zn_7_-MT (confirmed by ESI–MS). The pH of the protein was then adjusted to neutral pH.

Cu(I) titrations were carried out using either the apo MT or the ^68^Zn_7_-MT (formed as described earlier). When using apo MT, Cu(I) was added as tetrakis(acetonitrile)copper(I) hexafluorophosphate (Millipore-Sigma) in a 10 mM solution in 50% acetonitrile. When adding Cu(I) to the ^68^Zn_7_-MT, isotopically pure ^63^Cu(I) was used as this allows for the distinction between Cu(I) and Zn(II) by ESI–MS.^27^ Isotopically pure ^63^CuCl_2_ was purchased from Trace Sciences International. Glutathione (GSH) (Millipore Sigma) was used to reduce the copper from the 2+ oxidation state to the 1+ oxidation state as reported by Ferreira *et al*.^29^ GSH was dissolved in argon-saturated pH 7.4 10 mM ammonium formate and Cu(II) was added resulting in a 3:1 GSH: Cu(II) ratio. The mol. eq. of the reduced 10 mM Cu(I) solution (bound to GSH) were added to the ^68^Zn_7_-MT. The ESI–mass spectrum and emission spectrum of the result species were measured after a 10 min equilibration period. For each addition of ^63^Cu(I) mentioned, we mean the addition of Cu(I) bound to an unknown number of GSH and the oxidized glutathione (GSSH) product.

### ESI–MS methods

Samples were measured by direct infusion into the Bruker MicrOTOF II (Bruker Daltonics) in positive ion mode. The parameters used are shown in Table 1. All samples were rigorously deaerated and argon-saturated to prevent oxidation. The abundance of each species formed from the addition of Cu(I) to the protein was divided by the total recovered protein detected by the ESI–MS and multiplied by 100 to obtain a percentage abundance for each species. The percentage abundance of each species was plotted as a function of the Cu(I) bound to the protein.

**Table 1.** ESI–MS parameters

Parameter	Value
End plate offset	500 V
Capillary voltage	4500 V
Nebulizer	29.0 psi
Dry gas	6.0 L/min
Dry temp.	200°C
Target mass range	800–3000 m/z
Capillary exit voltage	180.0 V
Hexapole RF	600 Vpp

The ^63^Cu,^68^Zn-MT species distributions were determined by simulating the mass spectra of each possible species. The program mMass (version 5.5.0)^30^ was used to simulate the isotopic distribution of the apo protein. One or two protons were removed from the apo MT formula for each Cu(I) or Zn(II) bound to the protein, respectively, to mimic the deconvolution calculation where the charge of the metal ion is compensated for by proton loss. The mass of the appropriate number of ^68^Cu(I) and ^68^Zn(II) ions were added to the apo MT distribution. The mass spectra of species with the same total number of metals but differing Cu: Zn ratios were added in different fractions to identify the composition of the experimentally determined peak. Similar to the method used with the apo protein, the percentage abundance of each Cu, Zn-MT species was plotted as a function of the mol. eq. Cu(I) bound to the protein.

### Emission spectroscopy

The emission spectra were measured for the species forming in each step of the ^63^Cu(I) titration of Zn_7_-MT1A/2/3 using the Photon Technology International Quanta Master 4 scanning spectrophotometer (Photon Technology International, London, Ontario, Canada). Mass spectra were measured for the same solutions at each point in the titration to identify the species giving rise to the emission spectra. The MT solutions were kept in sealed quartz cuvettes with a septum to avoid oxidation of the protein and deactivation of the triplet excited state. The emission was stimulated by exciting the protein at 280 nm using a Xenon Flash lamp (flashing at 100 Hz) and the emission spectrum was measured from 500 to 900 nm. A yellow filter was used to eliminate scatter from the exciting beam. The emission slits were set to 10 nm and the exciting slits were set to 20 nm. The emission intensities were calibrated for the sensitivity of the GaAs phototube by measuring an HL-2000 HP Light Source (Ocean Insight, Orlando, FL, USA). The emission spectra at certain Cu(I) mol. eq. were compared for MT1A, MT2, and MT3.

### Binding constant simulations

The program Hyperquad Simulation & Speciation (HySS) was used to simulate a series of relative pH dependent, apparent cumulative binding constants, β, for the species forming upon the titration of apo MT1A, apo MT2, and apo MT3. The apparent β values were adjusted until the calculated speciation matched the experimental speciation from the ESI–mass spectral data. The apparent β values for undetected intermediate species were set at the highest values possible where they do not appear in the simulation. The pH dependent, apparent stepwise log K_F_ binding constants for species with n Cu(I) ions were calculated from the apparent log β values (log β*_n_*–log β*_n_*_-1_). Since HySS cannot handle the large β values that would result from the affinities expected for MT, only relative log β values are simulated. The apparent K_F_ values were then scaled so that the K_F_ for Cu_6_-MT was set to the value of Cu_6_-MT determined by Scheller *et al*. (also at pH 7.4).^26^

To demonstrate the unavoidable uncertainty when intermediate species are undetectable due to the cooperativity of certain species, simulations are shown that demonstrate the effect of changing the K_F_ of the unobserved species Cu_9_-MT2 vs. an observed species, Cu_10_-MT2.

## Results and discussion

### Cu(I) metallation of apo MT1A, apo MT2, and apo MT3

**Fig. 2:**
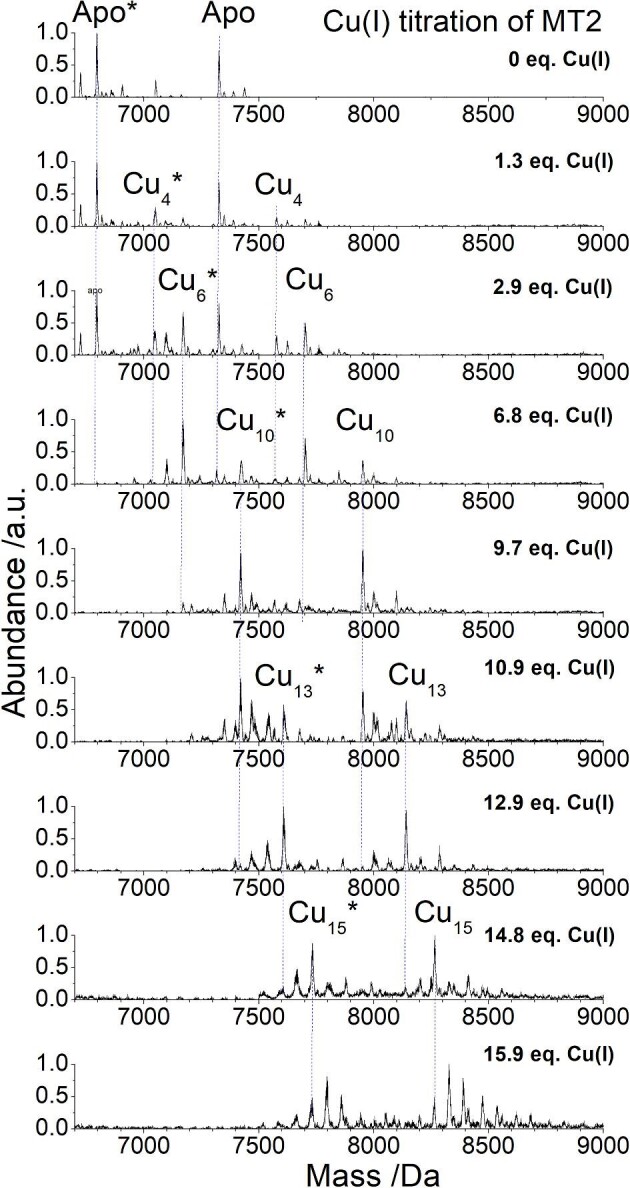
ESI–mass spectral data for the titration of Cu(I) into 50.1 μMapo MT2 at pH 7.4. The full MT2 protein is observed at 7325 Da in the mass spectrum. The main species forming after the addition of Cu(I) are Cu_4_-MT2, Cu_6_-MT2, Cu_10_-MT2, Cu_13_-MT2, and Cu_15_-MT2. A fraction of the protein has had the first “GSM” amino acids cleaved from the protein and is seen at 6795 Da (denoted Apo*). These amino acids are not part of the native protein sequence and do not affect metal binding. The Cu(I) metallation of this cleaved MT2 protein mirrors that of the full MT2 protein and the main species that form are Cu_4_*, Cu_6_*, Cu_10_*, Cu_13_*, and Cu_15_*. The masses of these species are 531 Da lower than the corresponding species in the full MT2 protein. The metal equivalences noted on the figure refer to the amount of Cu(I) bound to the protein.

Before comparing the more complicated speciation that arises from the addition of ^63^Cu(I) to ^68^Zn_7_-MT1A/2/3, we first compare the speciation that results from the addition of Cu(I) to apo MT1A,^31^ apo MT2, and apo MT3,^28^ which may occur in the body with newly synthesized MT. The ESI–mass spectral data for the titration of Cu(I) into apo MT2 are shown in Fig. 2. The titration closely resembles the Cu(I) titration of MT1A^26,31^; however, the Cu_15_-MT2 species is more prominent than in MT1A. Of note, Cu(I) titrations of rabbit liver MT revealed strong features in the circular dichroism (CD) spectra identified for a Cu_15_ species.^32^ Russell *et al*. reported similar species by ESI–MS after the addition of Cu(II). The authors reported that the use of the reducing agent, TCEP, would reduce the Cu(II) to Cu(I).^33^ We have not found the presence of TCEP to be sufficient in reducing Cu(II) to Cu(I) and so it is possible that some of the MT may have oxidized in that previous study. Conducting the Cu(I) titration of MT2 under the exact same conditions as we have reported for MT1A^31^ and MT3^28^ allows for a better comparison of the metallation properties.

**Fig. 3:**
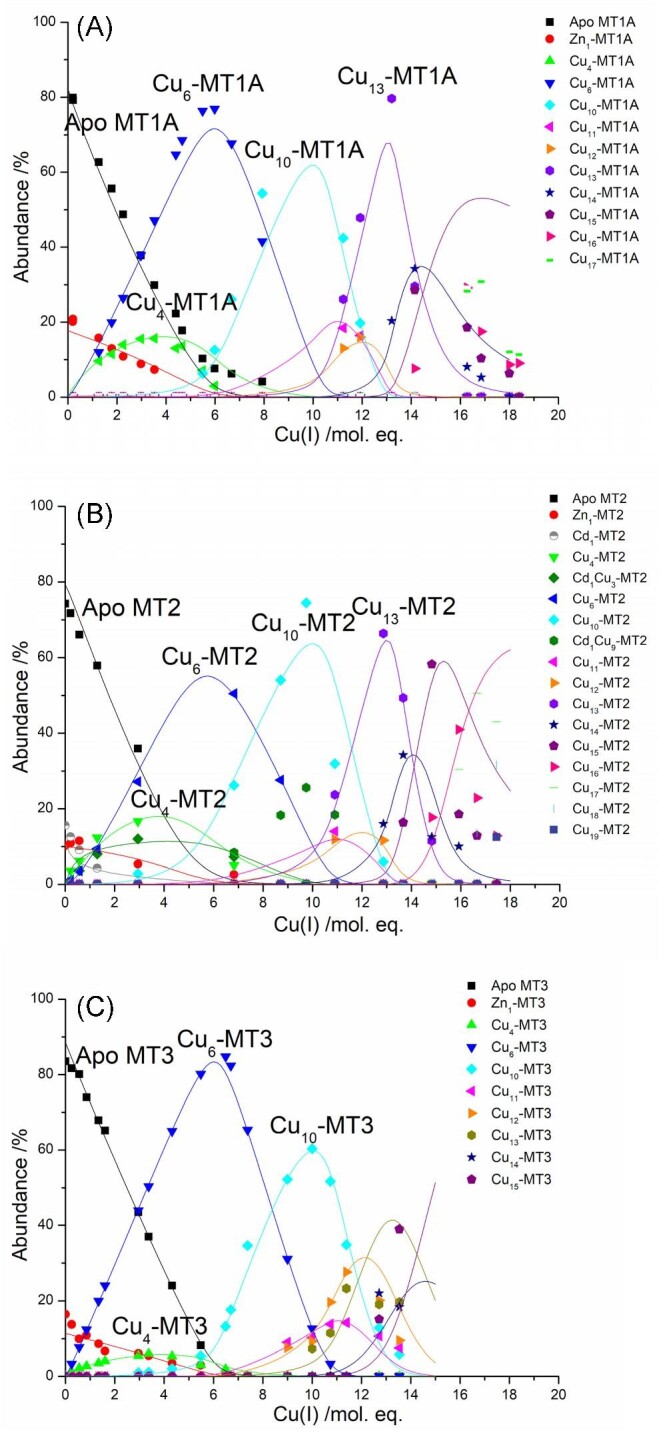
Speciation resulting from Cu(I) addition to apo MT1A, MT2, and MT3. (A) Experimental speciation (symbols) and simulated HySS speciation (lines) for Cu(I) binding to apo MT1A. Data originally published in Melenbacher *et al*.^31^ (B) Experimental speciation (symbols) and simulated HySS speciation (lines) for Cu(I) binding to apo MT2. (C) Experimental speciation (symbols) and simulated HySS speciation (lines) for Cu(I) binding to apo MT3. Data originally published in Melenbacher and Stillman.^28^ Log K_F_ values for key species shown in Fig. 6 and Table 3.

Figure 3 shows the abundance of each species forming during the titration of apo MT1A (Fig. 3A), apo MT2 (Fig. 3B) and apo MT3 (Fig. 3C) detected by ESI–MS as a function of the mol. eq. of Cu(I) bound to the protein. The speciation data were simulated using the software HySS. The titrations results for all three isoforms are similar with the formation of Cu_4_-MT1A/2/3, Cu_6_-MT1A/2/3, and Cu_10_-MT1A/2/3 in each protein at the approximately same Cu(I) loading.

The Cu_4_-MT1A/2/3 and Cu_6_-MT1A/2/3 species are proposed to have Cu_4_ and Cu_6_ clusters in the β domain. The Cu_10_-MT1A/2/3 species is a combination of a Cu_6_ cluster in the β domain and a Cu_4_ cluster in the α domain.^27,31^

The Cu(I) titration of apo MT3 differs significantly from the data for apo MT1A and apo MT2 in several respects. First, the relative abundance of the Cu_4_-species compared with the cooperatively formed Cu_6_-species for the MT3 is significantly less than observed for MT1 and MT2. Second, in MT1A and MT2, Cu_13_-MT1A/2 forms cooperatively after Cu_10_-MT1A/2, whereas in MT3, a series of species with increasing ratios of Cu(I) form non-cooperatively in low abundance with no species being preferred over the others. The Cu_13_-MT1A/2 species are proposed to be formed from a combination of a Cu_6_ cluster in the β domain and a Cu_7_ cluster in the α domain. The absence of a prominent Cu_13_-MT3 species suggests that the Cu_7_ cluster cannot form in the α domain of MT3, possibly due to the presence of the acidic loop, a hexapeptide insert of acidic amino acids (Fig. 1, residues 55–60). This insertion may destabilize the Cu_7_-α cluster. While it is possible that the Cu_13_-MT3 species specifically does not ionize as well as the Cu_13_-MT1A and Cu_13_-MT2 species or the prior Cu_10_-MT3 species, we have not found evidence for noticeable changes in ionization efficiencies when carrying out stepwise metallation of the same protein. In the past we have used emission spectroscopy,^31^ ultraviolet-absorption spectroscopy,^34,35^ and CD spectra^24^ as controls to confirm that the species ratios determined by the mass spectrometry fit the solution spectroscopic data.

**Fig. 4:**
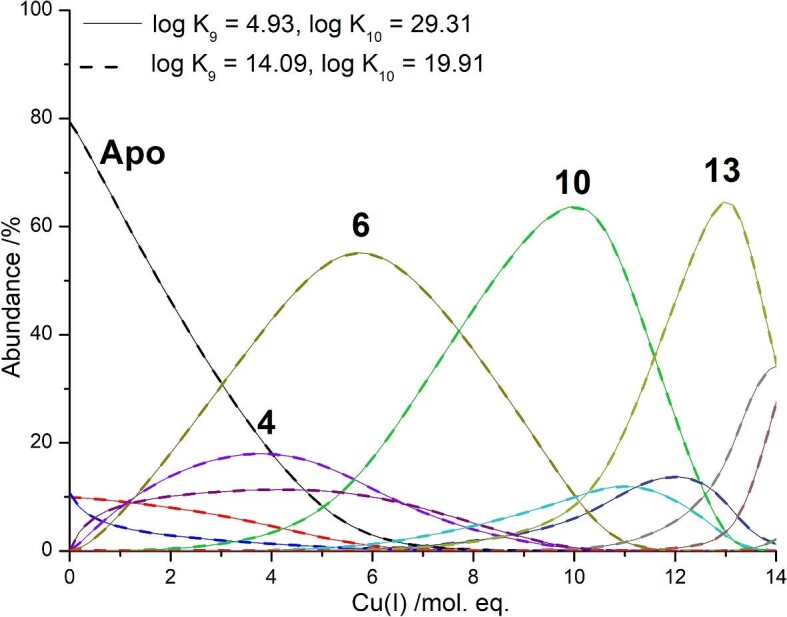
Simulated speciation for two values of log K_9_. Solid lines: Overall speciation if log K_9 _= 4.93 and log K_10 _= 29.31. Dashed lines: Overall speciation if log K_9 _= 14.09 and log K_10 _= 19.91. Remaining log K_F_ values are as reported in Table 3.

HySS was used to simulate relative pH dependent, apparent cumulative binding constants, log β, using a series of cumulative reactions (Table 2). The pH dependent, apparent K_F_ values for species with n Cu(I) ions for the stepwise reactions corresponding to the cumulative reactions shown in Table 2 were calculated by subtracting β*_n_*–β*_n_*_−1_. In the subsequent text and figures, all log β and log K_F_ values are the apparent constants determined at pH 7.4. We note that due to constraints with the program, the absolute values cannot be simulated and instead the resulting K_F_ values are scaled to literature values. The values of these log β were adjusted until the simulated speciation fit the experimental speciation (Fig. 3). It is chemically reasonable to predict that each metal binds one by one, though due to the cooperative properties of subsequent species, not all of the intermediate species form in detectable concentrations (the effect of a cooperative K_F_ of a species is to deplete the concentration of any species prior to the cooperative one). For all three isoforms, the β values for these intermediate species (i.e. Cu_1-3_-MT) which do not form in detectable amounts were set to the highest possible value before the species was evident in the simulation. Figure 4 demonstrates how log K_F_ of these unobserved species can be modified without significant impact on the overall speciation leading to much higher uncertainty in the log K_F_ values for these unobserved, but necessary, intermediates than for the observed species. With log K_9_ set between 4.93 (Fig. 4, solid line) and 14.09 (Fig. 4, dashed line), no detectable amounts of Cu_9_-MT2 form, as with the experimental data (Fig. 3B). Changing log K_9_ within this range has no impact of the species that form before and after, Cu_8_-MT2 and Cu_10_-MT2, respectively, as seen by the two sets of data lying on top of each other in Fig. 4. The effect of changing log K_9_ while leaving the remaining K_F_ values the same is variation in log K_10_ as log K_10_ is calculated by subtracting log β_9_ from log β_10_. While we demonstrate this variability in K_9_, we do however note that a log K_F_ of 4.93 is not reasonable for Cu(I) binding to thiols and we expect that the value is closer to the higher end of the spectrum. A log K_10_ of 19.91 agrees more readily with the published value for Cu_10_-MT, which was determined at pH 7.4 as well.^26^

**Table 2.** Series of cumulative and stepwise reactions used for determining binding constants

Cumulative Reaction	Apparent β	Stepwise Reaction	Apparent K_F_
apo MT_ _+ 1 Cu(I) ⇌ Cu_1_-MT	β_1_	apo MT_ _+ 1 Cu(I) ⇌ Cu_1_-MT	K_1_
apo MT_ _+ 2 Cu(I) ⇌ Cu_2_-MT	β_2_	Cu_1_-MT_ _+ 1 Cu(I) ⇌ Cu_2_-MT	K_2_
apo MT_ _+ 3 Cu(I) ⇌ Cu_3_-MT	β_3_	Cu_2_-MT_ _+ 1 Cu(I) ⇌ Cu_3_-MT	K_3_
apo MT_ _+ 4 Cu(I) ⇌ Cu_4_-MT	β_4_	Cu_3_-MT_ _+ 1 Cu(I) ⇌ Cu_4_-MT	K_4_
apo MT_ _+ 5 Cu(I) ⇌ Cu_5_-MT	β_5_	Cu_4_-MT_ _+ 1 Cu(I) ⇌ Cu_5_-MT	K_5_
apo MT_ _+ 6 Cu(I) ⇌ Cu_6_-MT	β_C6_	Cu_5_-MT_ _+ 1 Cu(I) ⇌ Cu_6_-MT	K_6_
apo MT_ _+ 7 Cu(I) ⇌ Cu_7_-MT	β_7_	Cu_6_-MT_ _+ 1 Cu(I) ⇌ Cu_7_-MT	K_7_
apo MT_ _+ 8 Cu(I) ⇌ Cu_8_-MT	β_8_	Cu_7_-MT_ _+ 1 Cu(I) ⇌ Cu_8_-MT	K_8_
apo MT_ _+ 9 Cu(I) ⇌ Cu_9_-MT	β_9_	Cu_8_-MT_ _+ 1 Cu(I) ⇌ Cu_9_-MT	K_9_
apo MT_ _+ 10 Cu(I) ⇌ Cu_10_-MT	β_10_	Cu_9_-MT_ _+ 1 Cu(I) ⇌ Cu_10_-MT	K_10_
apo MT_ _+ 11 Cu(I) ⇌ Cu_11_-MT	β_11_	Cu_10_-MT_ _+ 1 Cu(I) ⇌ Cu_11_-MT	K_11_
apo MT_ _+ 12 Cu(I) ⇌ Cu_12_-MT	β_12_	Cu_11_-MT_ _+ 1 Cu(I) ⇌ Cu_12_-MT	K_12_
apo MT_ _+ 13 Cu(I) ⇌ Cu_13_-MT	β_13_	Cu_12_-MT_ _+ 1 Cu(I) ⇌ Cu_13_-MT	K_13_
apo MT_ _+ 14 Cu(I) ⇌ Cu_14_-MT	β_14_	Cu_13_-MT_ _+ 1 Cu(I) ⇌ Cu_14_-MT	K_14_
apo MT_ _+ 15 Cu(I) ⇌ Cu_15_-MT	β_15_	Cu_14_-MT_ _+ 1 Cu(I) ⇌ Cu_15_-MT	K_15_

**Fig. 5:**
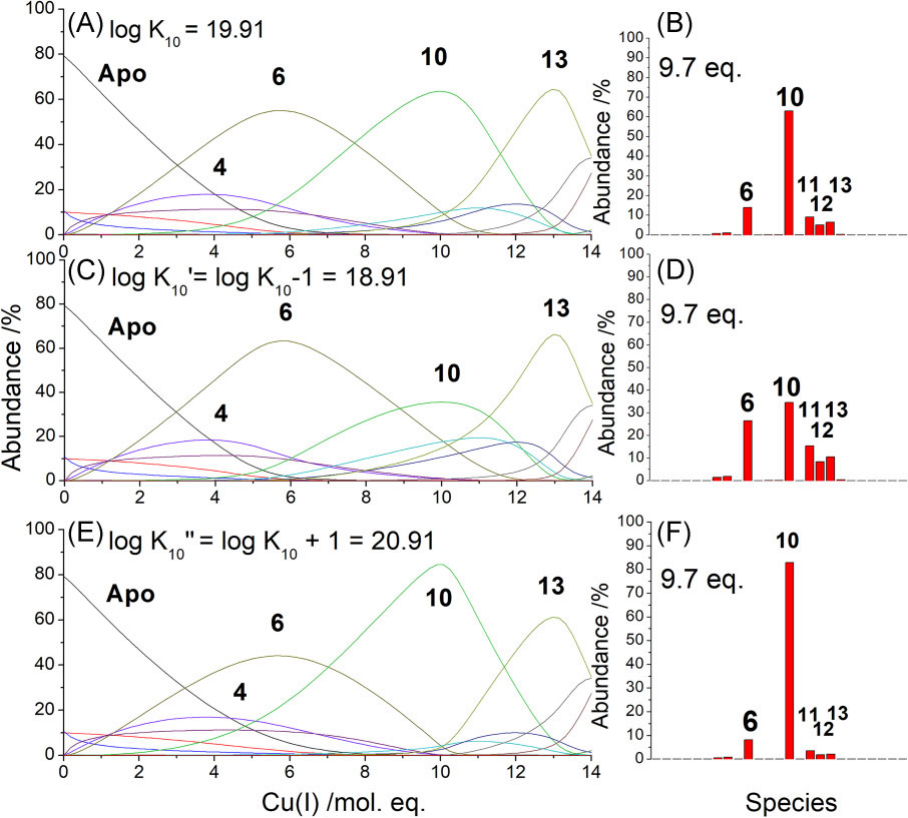
Simulated speciation and mass spectral profiles for varying log K_10_ values. (A) Simulated speciation where log K_10 _= 19.91, the value found to best simulate the experimental data in Fig. 3B. (B) Simulated mass spectral profile for the speciation in A with 9.7 mol. eq. Cu(I). (C) Simulated speciation where log K_10_ʹ = 18.91(i.e. log K_10_ − 1). (D) Simulated mass spectral profile for the speciation in C with 9.7 mol. eq. Cu(I). (E) Simulated speciation where log K_10_ʺ = 20.91 (i.e. log K_10 _+ 1). (F) Simulated mass spectral profile for the speciation in E with 9.7 mol. eq. Cu(I).

Whereas, adjusting the log K_F_ of one observed species, e.g. Cu_10_, by even just ±1 without changing any other of the other log K_F_ values, significantly impacts the speciation simulation, which results in different mass spectral profiles (Fig. 5). Figure 5A shows the speciation where log K_10__ _= 19.91, which is the value that fits the experimental data shown in Fig. 3B. The speciation shows that Cu_6_-MT2, Cu_10_-MT2, and Cu_13_-MT2 all reach a similar abundance throughout the titration. Figure 5B shows the mass spectral profile for this speciation with 9.7 mol. eq. Cu(I). The most abundant species in the spectrum is Cu_10_-MT2. Decreasing log K_10_ by 1 to 18.91 (log K_10_ʹ) results in significant changes to the speciation with Cu_10_-MT2 no longer reaching approximately the same abundance as Cu_6_-MT2 and Cu_13_-MT2 throughout the course of the titration. Now, Cu_10_-MT2 only reaches a maximum abundance of about 35% (Fig. 5C). Figure 5D shows the simulated mass spectral profile for this speciation with 9.7 mol. eq. Cu(I). Compared to Fig. 5D, if log K_10_ is decreased, then more Cu_6_-MT2 and Cu_11-13_-MT2 would be found in the mass spectrum. Figure 5E shows the resulting speciation if log K_10_ is increased by 1 to 20.91 (log K_10_ʺ). This results in the formation of much more Cu_10_-MT2 and much less Cu_6_-MT2 and, to a lesser extent, less Cu_13_-MT2 throughout the titration. Figure 5F shows how the mass spectral profile of this speciation with 9.7 mol. eq. Cu(I) has predominantly Cu_10_-MT2. Overall Figs. 4 and 5 show how the abundance of the observed species are much more sensitive to changes in the log K_F_ values than the unobserved intermediates that are assumed to form in the metallation pathway. This results in significantly greater errors in the log K_F_ values of those unobserved species in our model where the Cu(I) ions bind sequentially.

**Fig. 6:**
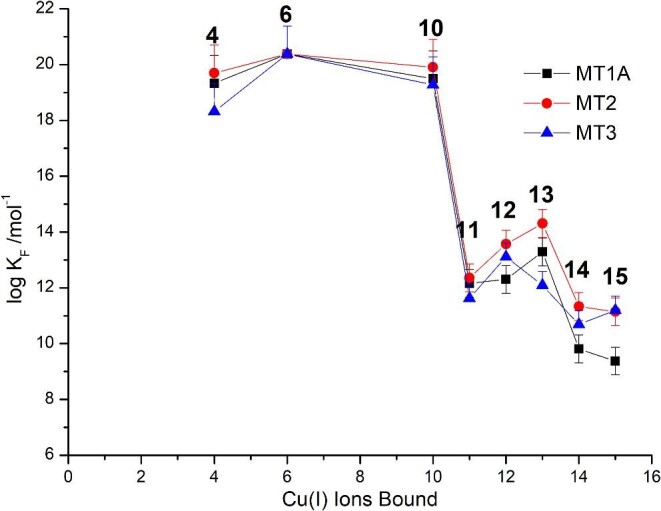
Log K_F_ values for observed species in the Cu(I) titration of MT1A (black squares), MT2 (red circles), and MT3 (blue triangles). Values listed in Table 3.

The HySS fits previously reported for MT1A^31^ and MT3^28^ were refit in the same manner so that the K_F_ values could be compared (Fig. 3). Based on the similarities in the formation of Cu_6_-MT in the speciation for all three isoforms at pH 7.4, the log K_F_ values were scaled so that the log K_F_ for Cu_6_ was 20.38, the value determined for MT1A by Scheller *et al*.^26^ at pH 7.4 (Fig. 6).

The general trend of the log K_F_ values is relatively similar for all three isoforms. There are slight differences in the log K_F_ for equivalent species forming in the three isoforms, which may be due to the error discussed earlier. The cooperativity of the Cu_4_-MT, Cu_6_-MT, and Cu_10_-MT is evident by the fact that the intermediates between these clustered species are not observed to form in the ESI–mass spectral data. Due to this, we have chosen not to show the log K_F_ values for these unobserved species in Fig. 6 as the error on the values is much higher than for the observed species as demonstrated earlier. To keep the analysis systematic between isoforms, the log β values used for the experimentally undetected species are the largest possible values that do not lead to detectable formation of that species. This may underestimate the values of log K_4_, K_6_, and K_10_. We report the log K_F_ values for the observed species up to Cu_15_-MT for MT1A, MT2, and MT3 in Table 3. We have added positive error bars on Fig. 6 for Cu_4_-MT, Cu_6_-MT, and Cu_10_-MT as these values are quite conservative due to the use of the largest possible log β values for Cu_3_-MT, Cu_5_-MT, and Cu_9_-MT. For Cu_11_-Cu_15_-MT, the error comes from the error in the fit, which we have indicated with smaller positive and negative error bars.

**Table 3.** Apparent log K_F_ values for Cu(I) binding to apo MT1A, apo MT2, and apo MT3

Cu_n_ (# of Cu(I) bound)	MT1A log K_F_ (M^−1^)^a^	MT2 log K_F_ (M^−1^)^a^	MT3 log K_F_ (M^−1^)^a^
4	19.33 ± 1	19.70 ± 1	18.32 ± 1
6	20.38 ± 1	20.38 ± 1	20.38 ± 1
10	19.49 ± 1	19.91 ± 1	19.27 ± 1
11	12.16 ± 0.5	12.36 ± 0.5	11.62 ± 0.5
12	12.30 ± 0.5	13.57 ± 0.5	13.11 ± 0.5
13	12.07 ± 0.5	14.30 ± 0.5	12.09 ± 0.5
14	8.91 ± 0.5	11.33 ± 0.5	10.69 ± 0.5
15	8.52 ± 0.5	11.14 ± 0.5	11.20 ± 0.5

a All log K_F_ values are apparent constants determined for pH 7.4.

In all three isoforms, the K_F_ values for Cu_6_-MT are higher than those for Cu_4_-MT and Cu_10_-MT, which explains the prominence of Cu_6_-MT early in the titration over Cu_4_-MT and Cu_10_-MT. Because the K_F_ for Cu_10_-MT is lower than that for Cu_6_-MT, there is only significant formation of Cu_10_-MT once all of the apo protein has been used to form Cu_6_-MT. In MT1A and MT2, the Cu_13_-MT1A/2 species forms with very high abundance compared to Cu_11_-MT1A/2, Cu_12_-MT1A/2, Cu_14_-MT1A/2 and Cu_15_-MT1A/2 (Fig. 3A, B). This is due to the K_F_ values increasing from Cu_11_ up to Cu_13_-MT1A/2 before dropping with Cu_14/15_-MT. In MT3, the K_F_ for Cu_12_-MT3 is higher than Cu_11_-MT3, Cu_13_-MT3, Cu_14_-MT3, and Cu_15_-MT3. However, the Cu_12_-MT3 species does not accumulate in MT3 to the same extent as Cu_13_-MT1A/2 due to the K_F_ values for Cu_13_-MT3, Cu_14_-MT3, and Cu_15_-MT3 being much more similar in magnitude compared to the difference between the K_F_ for Cu_13_-MT1A/2 and the K_F_ for Cu_14_-MT1A/2.

### Cu(I) metallation of Zn_7_-MT1A/2/3

We now turn to the Cu(I) metallation of Zn_7_-MT1A/2/3. We first compare the results of ^63^Cu(I) binding to ^68^Zn_7_-MT1A, ^68^Zn_7_-MT2, and ^68^Zn_7_-MT3^24,27,28^ before comparing our results to the speciation reported by others using different methods. The cationic charge introduced by the metals in these mixed Cu, Zn-MT species varies between 14+ and 17+ (Supplementary Fig. S17 in Melenbacher *et al.*^27^)

**Fig. 7:**
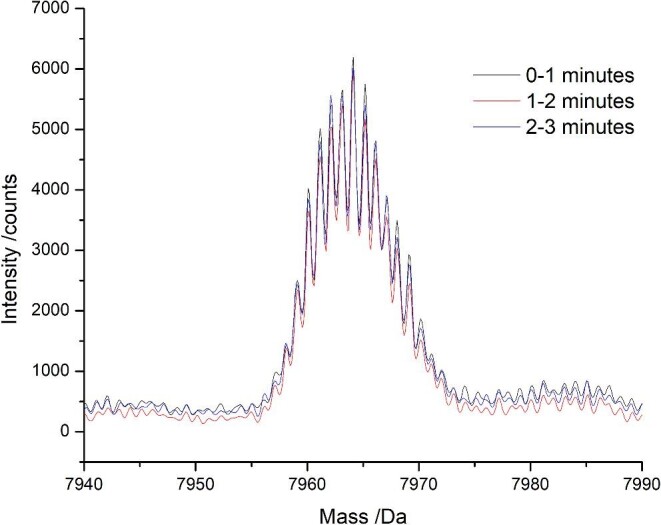
Deconvoluted Cu_5_Zn_5_/Cu_6_Zn_4_-MT2 peak calculated from the total ion chromatogram averaged over three separate times points.

**Fig. 8:**
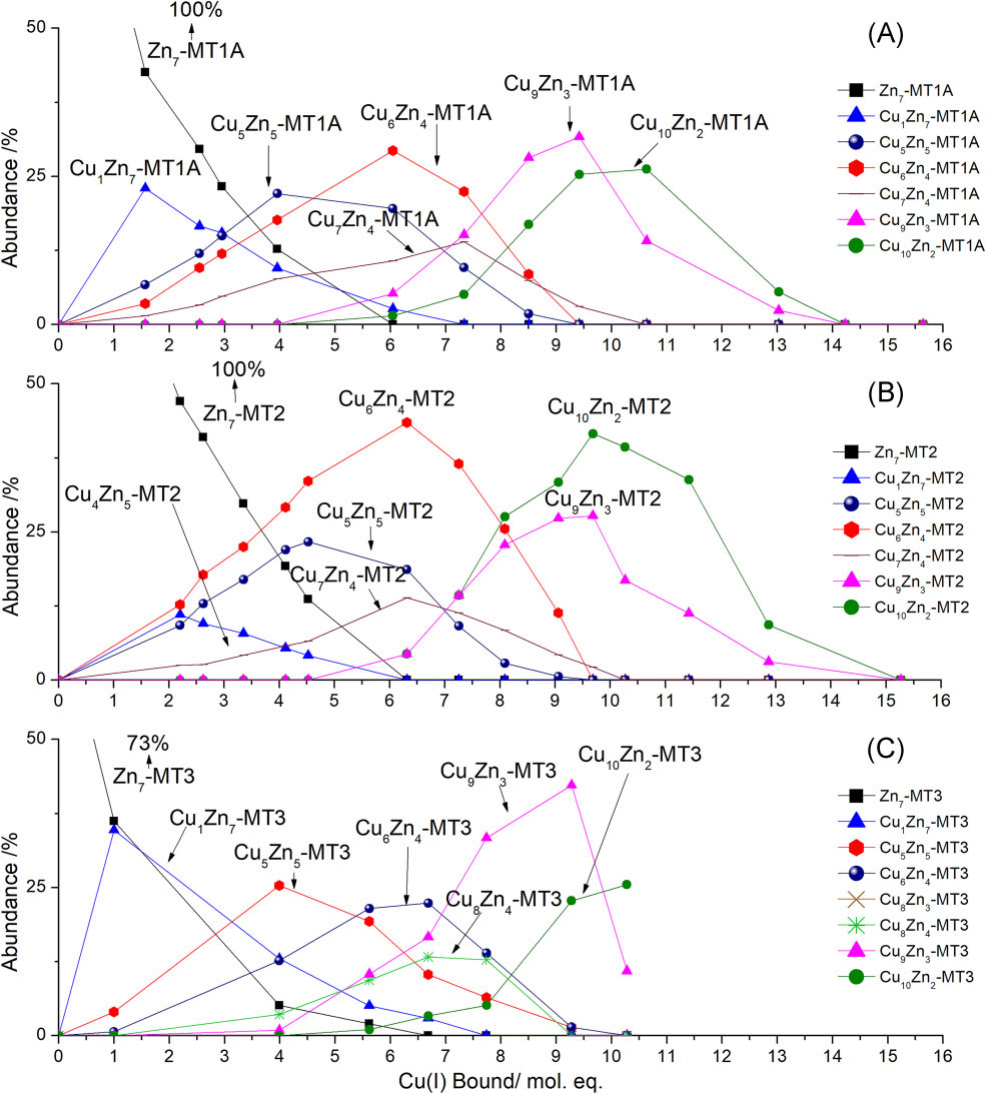
Speciation resulting from ^63^Cu(I) addition to ^68^Zn_7_-MT1A (A), ^68^Zn_7_-MT2 (B), and ^68^Zn_7_-MT3 (C) as determined from the corresponding ESI–mass m/z data (reported in Melenbacher *et al.*^27^ and Melenbacher and Stillman^24,28^). Only key species are shown for clarity. Full speciation shown in Supplementary Fig. S1.

The stability of the Bruker MicroTOF II ESI–MS was examined by integrating a chromatogram of a solution containing Cu_5_Zn_5_-MT2 and Cu_6_Zn_4_-MT2 over three different time points and comparing the deconvoluted spectrum. No change in the mass and very little change in the abundance were observed suggesting that the error of the mass spectral data is very small (Fig. 7). Figure 8 shows the speciation detected by ESI–MS after adding aliquots of ^63^Cu(I) to ^68^Zn_7_-MT1A (Fig. 8A), ^68^Zn_7_-MT2 (Fig. 8B), and ^68^Zn_7_-MT3 (Fig. 8C).

### Cu(I) metallation of each Zn_7_-MT isoform forms the Cu_1_Zn_7_-MT species first

The ESI–mass spectra reveal that the first species to form in MT1A, MT2, and MT3 is Cu_1_Zn_7_-MT (Fig. 8). This was unexpected as it was thought that the MT was saturated with seven divalent metals. Evidently, Cu(I) can bind to Zn_7_-MT without displacement of the Zn(II) which implies that the two domain structure set up by the Zn(II) has an available binding site for the Cu(I) ion in all three isoforms. It has not been determined where the Cu(I) ion is binding. It is possible that there are multiple structures for the Cu_1_Zn_7_-MT species. This significant result has implications in the formation of mixed metal species when low levels of Cu(I) exist with high cellular levels of Zn(II), because these data show that the Cu(I) can bind to the saturated Zn-MT.

**Fig. 9:**
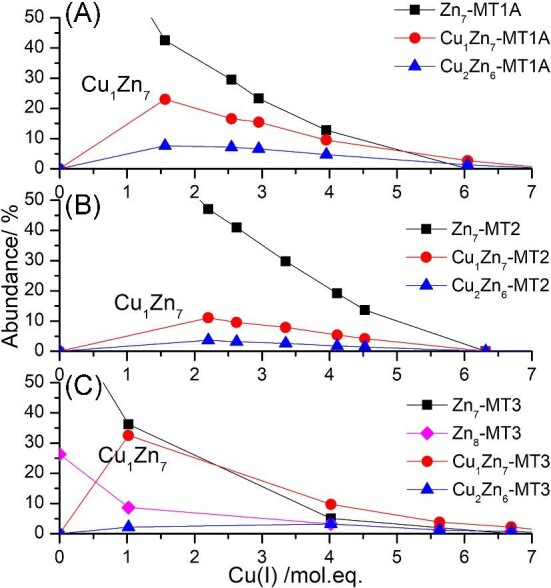
Abundance of Zn_7_-MT (black squares), Cu_1_Zn_7_-MT (red circles), and Cu_2_Zn_6_-MT (blue triangles) in MT1A (A), MT2 (B), and MT3 (C). Abundances calculated from ESI–MS data as first reported in Melenbacher *et al.*^27^ and Melenbacher and Stillman.^24,28^

It is only through the use of native ESI–MS methods, where all of the species in a solution can be disentangled, that the Cu_1_Zn_7_-MT species can be easily detected, unlike with other techniques that can only provide average stoichiometries. We note, as commented earlier, that we have no evidence for different metallated species of MT having different ionization efficiencies. Therefore, we expect the relative abundances of species detected in the ESI–mass spectra to align with the solution concentrations in these Cu(I) metallation studies. Figure 9 shows the correlation between the formation of the Cu_1_Zn_7_-MT and the loss of Zn_7_-MT in MT1A, MT2, and MT3. These data show that the fractions of the Cu_1_Zn_7_-MT formed are greatest for MT3, followed by MT1A, and then MT2. Palumaa *et al*. have reported that Cd_8_-MT3 and Zn_8_-MT3 form more readily compared the corresponding species in MT2.^22^ We have also observed the formation of Zn_8_-MT3 but not Zn_8_-MT1A or Zn_8_-MT2 (Fig. 9). We note that slight excess Zn(II) was added to apo MT to ensure that all of the protein became metallated with seven Zn(II) ions. With MT3, this resulted in the formation of a small amount of Zn8-MT3. The acidic loop in MT3 may allow for an eighth metal to bind more easily.

### The key species forming after Cu_1_Zn_7_-MT are Cu_5_Zn_5_-MT, Cu_6_Zn_4_-MT, Cu_9_Zn_3_-MT, and Cu_10_Zn_2_-MT in the three isoforms: MT1A, MT2, and MT3

Significantly, the stoichiometries for the dominant species forming in the next metallation steps after Cu_1_Zn_7_-MT in MT1A, MT2, and MT3 are essentially the same for the three isoforms with Cu_5_Zn_5_-MT1A/2/3, Cu_6_Zn_4_-MT1A/2/3, Cu_9_Zn_3_-MT1A/2/3, and Cu_10_Zn_2_-MT1A/2/3, all being prominent species (Fig. 8). An analysis of the relative binding constants for these species forming in MT2 revealed that these species form cooperatively. This is evident by the increase in the relative binding constants for these species compared to the previous species.^24^ Because of the similar speciation profiles, it is clear that the same is expected for the Cu(I) metallation of Zn_7_-MT1A and Zn_7_-MT3. As Cu(I) binds to the protein, the number of available binding sites decreases. This would result in a decrease in the relative binding constants due to the decreasing statistical likelihood of binding if the binding was non-cooperative or random.^31^ The speciation of non-cooperative binding is characterized by a binomial distribution of species centered on the average mol. eq. of the metal ion added, as evident by the completely non-cooperative binding of six As(III) ions to apo MT1A^35^ and apo MT3.^34^ With regards to MTs, we understand cooperativity as being the formation of clustered species, e.g. Zn_3_S_9_ and Zn_4_S_11_^36^ and in this paper Cu_6_S_9_(β)Zn_4_S_11_(α).

While the Cu, Zn-MT species that form are similar for MT1A,^27^ MT2,^24^ and MT3,^28^ there are differences in the relative abundances of the species resulting in different overall metallation profiles. The main difference is the prominence of the Cu_1_Zn_7_-MT, Cu_6_Zn_4_-MT, and Cu_10_Zn_2_-MT species.

### Analysis of MT1A, MT2, and MT3 speciation through ESI–MS led to the determination of multiple separate pathways for stepwise Cu(I) binding

**Scheme 1:**
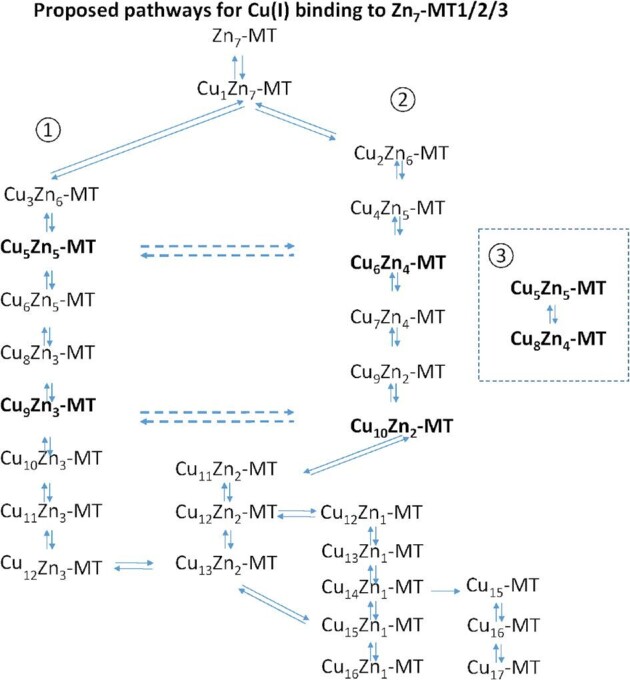
Proposed pathway of species forming from human Zn_7_-MT for MT1A, MT2, and MT3 adapted from the pathway proposed for MT2 by Melenbacher and Stillman.^24^

**Fig. 10:**
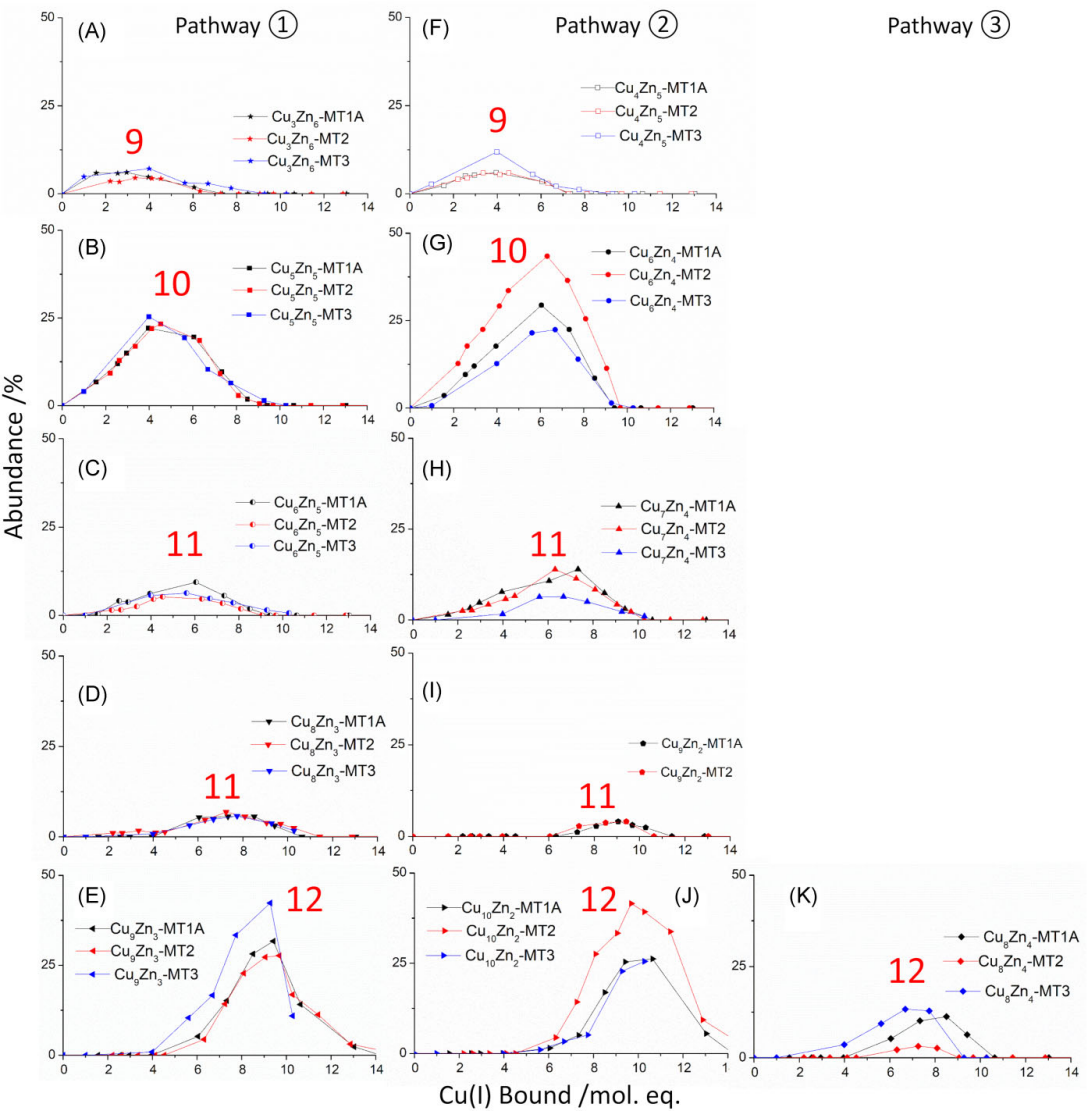
Comparison of species abundance for each of MT1A, MT2, and MT3 for the three proposed pathways. Abundance of each of the species detected through ESI–MS as shown in Melenbacher *et al.*^27^ and Melenbacher and Stillman.^24,28^ Red numbers indicate the total number of metals bound to the protein. (A–E) Pathway 1 species formed in MT1A (black), MT2 (red), and MT3 (blue). (F–J) Pathway 2 species formed in MT1A (black), MT2 (red), and MT3 (blue). (K) Pathway 3 species formed in MT1A (black), MT2 (red), and MT3 (blue). Isoform-dependent differences in the abundance of Zn_7_-MT, Cu_1_Zn_7_-MT, and Cu_2_Zn_6_-MT shown in Fig. 9.

The coexistence of two pairs of species with the same total number of metals bound (10 metals in Cu_5_Zn_5_-MT2 and Cu_6_Zn_4_-MT2 and 12 metals in Cu_9_Zn_3_-MT2 and Cu_10_Zn_2_-MT2) led us to propose two parallel pathways in our detailed analysis of the speciation for Cu, Zn-MT2. Cu(I) metallation pathway ① forms Cu_5_Zn_5_-MT2 and Cu_9_Zn_3_-MT2 and pathway ② forms Cu_6_Zn_4_-MT2 and Cu_10_Zn_2_-MT2.^24^ The presence of this same series of species in MT1A and MT3 allow us to now propose that Cu(I) metallation follows similar pathways in these isoforms as well (Scheme 1). The bolded species are the major species of each pathway and the non-bolded species are proposed intermediates. Comparison of the speciation for MT1A, MT2, and MT3 reveals that certain species form with remarkably similar abundances over the same range of Cu(I) bound (Fig. 10). By focusing on one species at a time, multiple pathways can be suggested for each of MT1A, MT2, and MT3. We note that the identification of multiple pathways is only possible with the use of isotopically pure ^63^Cu(I) and ^68^Zn(II) as this allows us to distinguish species from separate pathways with the same total number of metals, but differing Cu: Zn ratios (ex. Cu_5_Zn_5_-MT and Cu_6_Zn_4_-MT).

### The significant role of Cu_1_Zn_7_-MT

We propose that after the formation of Cu_1_Zn_7_-MT, subsequent Cu(I) metallation can follow one of several pathways. The fact that this species branches off into multiple pathways might mean that there are multiple structures for the initial Cu_1_Zn_7_-MT species.

**Pathway ①** The first species to form from Cu_1_Zn_7_-MT in pathway ① is Cu_3_Zn_6_-MT (Fig. 10A). The exchange of one Zn(II) ion for two Cu(I) ions conserves the charge of the molecule. It was seen in MT1A that the species forming all contain a cationic charge from the metal within a certain range.^27^ The replacement of one Zn(II) ion by two Cu(I) ions is seen again with the formation of Cu_5_Zn_5_-MT (Fig. 10B) from Cu_3_Zn_6_-MT. The distribution and abundance of the Cu_5_Zn_5_-MT remains constant with all three isoforms. Next, we propose that one Cu(I) ion binds to Cu_5_Zn_5_-MT to form Cu_6_Zn_5_-MT (Fig. 10C) and then two Cu(I) ions replace two Zn(II) ions to form Cu_8_Zn_3_-MT (Fig. 10D). The next major product to form in pathway ① is Cu_9_Zn_3_-MT (Fig. 10E). All of the species appearing in pathway ① form with approximately equal abundances across MT1A, MT2, and MT3. This indicates that the relative binding constants (K_F_) for the species in pathway ① are approximately the same for these structures across all three MT isoforms.

**Pathway ②** While the abundances of the species forming in pathway ① remain relatively constant across MT1A, MT2, and MT3, the species forming in pathway ② show isoform-dependent differences indicating different relative K_F_ values for different human MT isoforms. We show in Fig. 5 that the value of a stepwise binding constant, K_F_, relative to the other species affects the relative abundance of a species. We propose that the first product of pathway ② is Cu_6_Zn_2_-MT (Fig. 9). We propose that the next species to form in pathway ② is Cu_4_Zn_5_-MT (Fig. 10F). Equal fractions of Cu_4_Zn_5_-MT1A and Cu_4_Zn_5_-MT2 form with slightly higher fractions of Cu_4_Zn_5_-MT3 forming.

The next species proposed to form from this pathway is Cu_6_Zn_4_-MT (Fig. 10G). The abundance of this species is highest in MT2, followed by MT1A, and then MT3. This suggests that the K_F_ for Cu_6_Zn_4_-MT formation follows the trend of MT2 > MT1A > MT3. We note here that because the formation of the Cu(I) is through reduction by GSH, there will be excess GSH over the µM concentration of the Cu(I) added. This GSH will compete for the Zn(II) that is released from the MT as a result of Cu(I) binding.^37^ This competition becomes more significant with higher ratios of Cu(I) bound, but under those conditions, the Cu(I) binding dominates. The experimental evidence from our data is that the GSH does not exert a significant impact because even with 14 Cu(I) ions, one Zn(II) ion remains bound to MT, which we would have expected to be removed by the GSH.

Cu(I) binding to Cu_6_Zn_4_-MT results in Cu_7_Zn_4_-MT (Fig. 10H). This species forms in similar abundances in MT1A and MT2. Slightly less Cu_7_Zn_4_-MT forms in MT3. This species is consumed to form Cu_9_Zn_2_-MT (Fig. 10I). The next species to form after Cu_9_Zn_2_-MT is Cu_10_Zn_2_-MT (Fig. 10J). Approximately equal amounts of Cu_10_Zn_2_-MT forms in MT1A and MT3, indicating that the binding constants for the two isoforms of this species are similar. There is an increase in the amount of Cu_10_Zn_2_-MT2 that forms, which suggests that its K_F_ is slightly higher than for MT1A and MT3.

**Pathway ③** We propose that Cu_8_Zn_4_-MT (Fig. 10K) may be part of a third pathway. Based on data from Zn_7_-MT3 carried out at pH 7.8, the Cu_8_Zn_4_-MT may form from Cu_5_Zn_5_-MT3.^28^

### MT isoforms show different preferences for pathways

**Fig. 11:**
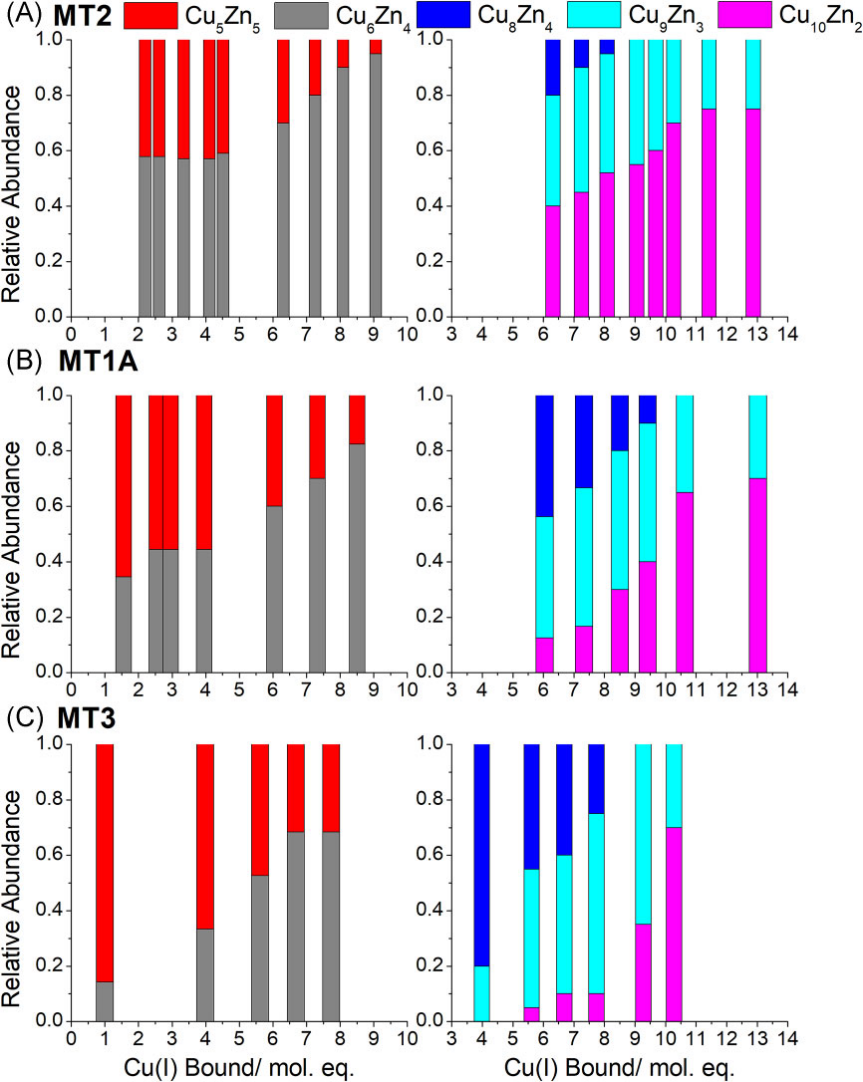
Fraction of different M_10_ and M_12_ species forming in MT1A, MT2, and MT3 as a function of Cu(I) bound to the protein. Fractions determined from the ESI–mass spectral data reported in Melenbacher *et al.*^27^ and Melenbacher and Stillman.^24,28^ (A) Left: Fraction of Cu_5_Zn_5_-MT2 (red) and Cu_6_Zn_4_-MT2 (gray). Right: Fraction of Cu_8_Zn_4_-MT2 (blue), Cu_9_Zn_3_-MT2 (cyan), and Cu_10_Zn_2_-MT2 (pink). (B) Left: Fraction of Cu_5_Zn_5_-MT1A (red) and Cu_6_Zn_4_-MT1A (gray). Right: Fraction of Cu_8_Zn_4_-MT1A (blue), Cu_9_Zn_3_-MT1A (cyan), and Cu_10_Zn_2_-MT1A (pink). (C) Left: Fraction of Cu_5_Zn_5_-MT3 (red) and Cu_6_Zn_4_-MT3 (gray). Right: Fraction of Cu_8_Zn_4_-MT3 (blue), Cu_9_Zn_3_-MT3 (cyan), and Cu_10_Zn_2_-MT3 (pink).

The ratio between the 10 metal species (M_10_) species, Cu_5_Zn_5_-MT and Cu_6_Zn_4_-MT, is different for MT1A, MT2, and MT3 (Fig. 11, left). These ratios indicate which pathways are most prevalent in the three isoforms of human MT and provide guidance as to metal-binding preferences at the cellular level. As noted earlier, pathway ① forms Cu_5_Zn_5_-MT (Fig. 11, red bars) and Cu_9_Zn_3_-MT (Fig. 11, cyan bars). Pathway ② results in Cu_6_Zn_4_-MT (Fig. 11, gray bars) and Cu_10_Zn_2_-MT (Fig. 11, pink bars). Pathway ③ results in Cu_8_Zn_4_-MT (Fig. 11, blue bars). The left side of Fig. 11 shows that, of the M_10_ species, Cu_6_Zn_4_-MT2 (pathway ②) forms in a higher ratio compared to Cu_5_Zn_5_-MT2 with greater than 2 mol. eq. Cu(I) bound. This is unlike the Cu(I) titration of Zn_7_-MT1A and Zn_7_-MT3. With MT1A (Fig. 11B, left) and MT3 (Fig. 11C, left), the M_10_ species forms initially as Cu_5_Zn_5_-MT with the abundance of Cu_6_Zn_4_-MT surpassing Cu_5_Zn_5_-MT only after approximately 5.0 mol. eq. Cu(I) bound to Zn_7_-MT1A or Zn_7_-MT3. This indicates that MT2 has more of a preference for pathway ②, even with low Cu(I), compared to MT1A and MT3. This preference for Cu_6_Zn_4_-MT2 of pathway ② over Cu_5_Zn_5_-MT2 formed from pathway ① may be due to Zn(II) binding to the β domain of MT2 with weaker affinity compared to MT1A or MT3, or Cu(I) binding stronger compared to MT1A and MT3.

The ratio between the M_12_ species formed in MT2 (Fig. 11A, right) also differs from those formed in MT1A (Fig. 11B, right) and MT3 (Fig. 11C, right). In MT2, Cu_10_Zn_2_-MT2 forms in higher abundance compared to the other M_12_ species, which is consistent with MT2 preferring pathway ②. Whereas with MT1A^27^ and MT3,^28^ the Cu_8_Zn_4_-MT and Cu_9_Zn_3_-MT species first formed in greater abundance compared to Cu_10_Zn_2_-MT. With between 6 and 9 mol. eq. Cu(I) bound, metallation of Zn_7_-MT1A/3 preferentially goes through pathway ① to form Cu_9_Zn_3_-MT1A/3.

### Emission spectra indicate similar Cu(I)-cysteine cluster formation in MT1A, MT2, and MT3

**Fig. 12:**
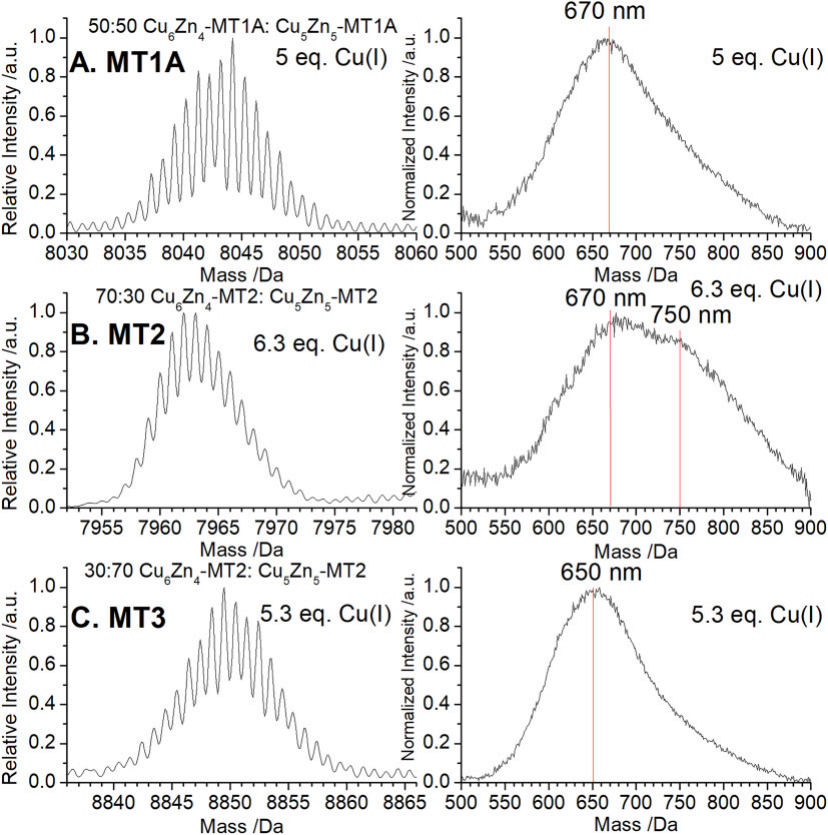
ESI–mass spectra and emission spectra of Cu_5_Zn_5_-MT and Cu_6_Zn_4_-MT forming following stepwise Cu(I) metallation of Zn_7_-MT1A, Zn_7_-MT2, and Zn_7_-MT3.The *x*-axis for the mass spectra shows a 60 Da range centered on the mass of Cu_6_Zn_4_-MT for each of MT1A, MT2, and MT3. (A) ESI–mass spectrum (left) and emission spectrum (right) for 5.0 mol. eq. Cu(I) bound to Zn_7_-MT1A. (B) ESI–mass spectrum (left) and emission spectrum (right) for 6.3 mol. eq. Cu(I) bound to Zn_7_-MT2. (C) ESI–mass spectrum (left) and emission spectrum (right) for 5.3 mol. eq. Cu(I) bound to Zn_7_-MT3. Data originally reported in Melenbacher *et al.*^27^ and Melenbacher and Stillman.^24,28^

We show that the emission bands are dependent on the cluster stoichiometry through parallel ESI–MS and emission experiments on MT1A, MT2, and MT3. Species with the same Cu: Zn stoichiometry have emission spectral bands at similar emission wavelengths, regardless of the MT isoform. Figure 12 shows the ESI–mass spectra indicating the presence of varying ratios of Cu_5_Zn_5_-MT and Cu_6_Zn_4_-MT (left) and the corresponding emission spectra (right) for these species in MT1A (Fig. 12A), MT2 (Fig. 12B), and MT3 (Fig. 12C). The emission at 650–670 nm is attributed to the Cu_5_Zn_5_-MT species and the emission with a band center at approximately 750 nm is from the Cu_6_Zn_4_-MT. The relative intensity of the 750 nm band correlates with the abundance of Cu_6_Zn_4_-MT found in the ESI–mass spectral data. The similarity in the emission wavelengths confirm the very high reliability of the solution-state emission in identifying the presence of specific Cu_n_S_m_ clusters. The slight shift in the wavelength of the Cu_5_Zn_5_-MT3 cluster compared to the equivalent cluster in MT1A and MT2 may be due to distortions in the cluster caused by the CPCP motif found only in MT3. It is only by combining the room temperature solution phosphorescence measurements with the ESI–mass spectral measurements can the stoichiometries of the clusters giving rise to the emission be determined. The emission spectra are actually more sensitive to the presence of multiple species as the ESI–mass spectra can only be disentangled with the use of both the isotopically pure metals and mass spectral simulations.

**Fig. 13:**
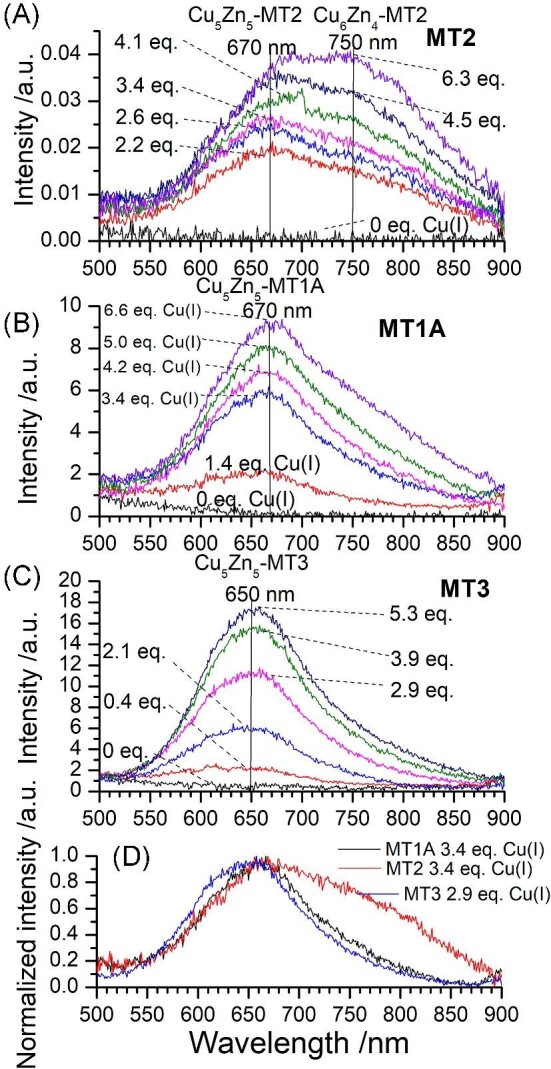
Room temperature emission for the addition of ^63^Cu(I) to ^68^Zn_7_-MT1A/2/3. (A) Emission spectra measured for the addition of ^63^Cu(I) to ^68^Zn_7_-MT2 at pH 7.4. (B) Emission spectra measured for the addition of ^63^Cu(I) to ^68^Zn_7_-MT1A at pH 7.4. (C) Emission spectra measured for the addition of ^63^Cu(I) to ^68^Zn_7_-MT3 at pH 7.8. (D) Normalized emission spectra for MT1A (black), MT2 (red), and MT3 (blue) when 2.9–3.4 mol. eq. Cu(I) is bound to the protein. Data originally reported in Melenbacher *et al.*,^27^ and Melenbacher and Stillman.^24,28^

Figure 13 shows the emission profiles for the first six Cu(I) ions bound to Zn_7_-MT for MT2 (Fig. 13A), MT1A (Fig. 13B), and MT3 (Fig. 13C). While the emission wavelengths are similar for MT1A,^27^ MT2,^24^ and MT3,^28^ there are differences within the first six mol. eq. Cu(I) bound that reflect the different ratios of M_10_ species forming. All three isoforms show an increase in the emission at 650/670 nm due to the formation of Cu_5_Zn_5_-MT. The formation of Cu_6_Zn_4_-MT can be tracked through the presence of the 750 nm emission. The similarities in the emission wavelengths indicate that the excited state properties of the clusters are relatively similar across MT1A, MT2, and MT3.

MT2 exhibits the greatest intensity of the 750 nm emission, followed by MT1A, and finally MT3. This follows the trends in the ESI–mass spectral data for the formation of Cu_6_Zn_4_-MT vs. Cu_5_Zn_5_-MT (Fig. 8). Figure 13D shows the emission spectra resulting from of 3.4 mol. eq. Cu(I) bound to Zn_7_-MT1A (Fig. 13D, black), 3.4 mol. eq. Cu(I) bound to Zn_7_-MT2 (Fig. 13D, red), and 2.9 mol. eq. Cu(I) bound to Zn_7_-MT3 (Fig. 13D, blue). It is clear in the emission that MT2 forms Cu_6_Zn_4_-MT very early in the titration and in greater fractions compared to MT1A and MT3. These emission spectra support the ESI–MS data that show the formation of Cu_6_Zn_4_-MT through pathway ② is preferred in MT2 more than MT1A and MT3. The emission spectra for the remainder of the titration are very similar to MT1A and MT3.

### MT isoforms do not show signs of being optimized for different metals

Analysis of ESI–mass spectral data allows for novel comparisons of the species forming in human MT1A, MT2, and MT3 after the addition of Cu(I) to the apo or Zn_7_ forms of the proteins. These conclusions concerning speciation are supported by the room temperature Cu_n_S_m_ cluster-dependent emission spectra. A series of similar Cu(I)-thiolate clusters form following the stepwise addition of Cu(I) to the apo proteins of all three isoforms. The species forming when ^63^Cu(I) is added to ^68^Zn_7_-MT1A, ^68^Zn_7_-MT2, and ^68^Zn_7_-MT3 are more complicated. The first species to form is Cu_1_Zn_7_-MT and then a series of species with the same stoichiometries form following one of three separate pathways for each of the three isoforms. Despite the differences in the non-cysteine amino acids between the three isoforms, our data suggest that it is the conserved positions of the cysteines that largely control the exact speciation. However, there are subtle differences in the abundance of some species. This indicates that there are slight isoform-dependent differences that control the adopted metallation pathway though all three pathways exist in each isoform. Even with these pathway differences, we find no evidence for Cu(I) binding being optimized for one MT isoform over the other when starting with either the apo or Zn_7_ form of MT1A, MT2, and MT3. While the data show a lack of Cu_13_-MT when Cu(I) binds to apo MT3 unlike MT1A and MT2, we do not expect this difference to be significant *in vivo* where additional metals would induce the synthesis of additional MT, effectively decreasing the metal: MT ratio.

Our conclusions differ significantly from those reported for mouse MT1, MT2, and MT3.^19,23^ While our systematic comparison of Cu(I) binding to Zn_7_-MT1A, Zn_7_-MT2, and Zn_7_-MT3 examines the binding properties and species formation *in vitro*, the methodology used in the mouse MT studies involved synthesizing recombinant MT in the presence of excess Cd(II), Zn(II), or Cu(I) in the *E. coli* overexpression system before isolating and purifying the protein from that cellular milieu. Those authors concluded that mouse MT2 was not optimized for Cu(I) coordination after experiencing difficulty isolating Cu(I) containing MT2. As a result, Atrian *et al*. labeled mouse MT2 as a Zn-thionein.^19^ We would expect that if MT2 was truly less optimized for Cu(I) than the other isoforms that we would see different species forming when Cu(I) was added to the apo or Zn_7_ forms of the protein compared to MT1A and MT3 and so we conclude that MT2 is no less optimized for binding Cu(I) than MT1A or MT3. The binding of Cu(I) to Zn_7_-MT2 is actually more cooperative than for Zn_7_-MT1A and Zn_7_-MT3 as seen by the preferential formation of Cu_6_Zn_4_-MT2 over Cu_5_Zn_5_-MT2 compared to MT1A and MT3. Our group has also previously published Cu(I) binding studies of rabbit liver MT2^25,38^ suggesting that this is not just a property of human MT. The differences found in those previous reports by others may be explained by difficulties in purifying Cu-MTs, a very air sensitive form of MT, from the *E. coli*. Contrasting results to the study by Atrian *et al*.^19^ were also found by Sakurai *et al*.^39^ This study separated MT isoforms 1 and 2 isolated from the livers of rats using polyacrylamide-coated capillary zone electrophoresis. The study analysed the MT content from livers of Long–Evans cinnamon (LEC) rats, an animal model of Wilson's disease where Cu(I) accumulates in the liver, as well as control rats exposed to CdCl_2_. Whereas Cu(I) accumulation in the livers of the LEC rats led to an increase in the concentration of MT2 compared to MT1, the accumulation of Cd(II) in the livers of the control rats led to an increase in the concentrations of MT1 compared to MT2.^39^ These differences are likely a result of different gene expression rather than the intrinsic capability of a MT isoform to bind a specific metal. These results suggest that MT2 is preferred for the chelation of excess Cu(I) over MT1A.^19^ While our results do show the preference of Cu_6_Zn_4_-MT2 over the same stoichiometries in MT1A and MT3, we do not believe that this difference is large enough to justify characterizing MT2 as more of a “Cu-MT” than the other human MTs.

This brings us to the question “Cu(I) and Zn(II) bind to MT in cells, but what is known about that stoichiometric ratio?” Under normal Cu(I) and Zn(II) levels, what would we expect the stoichiometry to be? We propose that expression of MT in the presence of Cu(I) and Zn(II) will form a number of species dominated by the cooperative formation of the Cu(I) clusters. Our results combined with results of MTs isolated from tissues^6,9,12,14,15^ suggest a mixture of Cu(I) and Zn(II) under normal physiological conditions and that the ratio of Cu: Zn bound to MT varies.^9^ We have seen that Cu(I) first replaces the Zn(II) from the β domain to cooperatively form clusters of five to six Cu(I) ions suggesting that with low Cu(I) conditions *in vivo* (i.e. less than six), the β domain is likely to contain Cu(I) whereas the α domain will contain varying amounts of Zn(II),^24^ depending on the starting Zn(II) levels. This may be how MT specifically metallates other enzymes with Cu(I) vs. Zn(II). A major result of the ESI–mass spectral studies that is not replicated in any other method is quantitation of all species in solution and the evidence that multiple species can coexist for a specific Cu: Zn: MT ratio.

## Conclusions

The species forming in MTs following the addition of Cu(I) are complicated and ESI–mass spectral analysis is required to analyse the detailed metallation properties. The species forming after the addition of Cu(I) to apo MT1A, MT2, and MT3 were compared and the relative binding constants for Cu_4_-MT, Cu_6_-MT, and Cu_10_-MT were determined for all three isoforms. The use of ^63^Cu(I) and ^68^Zn(II) has allowed for the determination of exact Cu: Zn ratios for all species forming after the addition of ^63^Cu(I) to ^68^Zn_7_-MT1A/2/3. The species forming in MT1A, MT2, and MT3 were compared to determine whether there are isoform-dependent differences in Cu(I) binding. Strikingly, species with the same stoichiometry form in MT1A, MT2, and MT3. All three MT isoforms exhibited similar room temperature emission spectra with wavelengths dependent on the MT stoichiometry. The combination of different emission bands gave insight into the mixtures of species forming. Analysis of the species revealed three pathways with pathways ① and ② being the prominent pathways in all three isoforms. Pathway ① results in Cu_5_Zn_5_-MT and Cu_9_Zn_3_-MT. Pathway ② results in Cu_6_Zn_4_-MT and Cu_10_Zn_2_-MT. Pathway ③ results in Cu_8_Zn_4_-MT. Both the ESI–mass spectral results and room temperature emission spectra indicate that pathway ② is preferred most with MT2, followed by MT1A, and then MT3. Unlike other reports, we do not find significant differences in the Cu(I) binding abilities for the three MT isoforms tested suggesting that if the MT isoforms do have different functions, it is due to differences in gene regulation and expression rather than their metal binding abilities.

## Supplementary Material

mfae015_Supplemental_File

## Data Availability

The data underlying this article are available in the article or are available in the cited publications.
