# Supplementary material for: ESI–MS analysis of Cu(I) binding to apo and Zn7 human metallothionein 1A, 2, and 3 identifies the formation of a similar series of metallated species with no individual isoform optimization for Cu(I)
Source: Metallomics. 2024 Mar 19;16(4):mfae015. doi: 10.1093/mtomcs/mfae015 (PMC11004924; doi:10.1093/mtomcs/mfae015)
Supplement: mfae015_Supplemental_File [file mfae015_supplemental_file.docx]

**ESI-MS analysis of Cu(I) binding to apo and Zn_7_ human metallothionein 1A, 2, and 3 identifies the formation of a similar series of metallated species with no individual isoform optimization for Cu(I): Supplementary Information**

Adyn Melenbacher^a^, Martin J. Stillman^*a^

^a^Department of Chemistry, The University of Western Ontario, London, ON, Canada

**Corresponding Author**

* Martin J. Stillman. Department of Chemistry, The University of Western Ontario, 1151 Richmond St., London, ON, Canada, N6A 5B7

Running Title: MT1A/2/3 have similar Cu metallation properties

**ABBREVIATIONS**

CD, circular dichroism; ESI-MS, electrospray ionization mass spectrometry; *E. coli, Escherichia coli*; GSH, glutathione; GSSH, oxidized glutathione; HySS, Hyperquad Simulation & Speciation; LMCT, ligand to metal charge transfer; LEC, Long-Evans Cinnamon; MT, metallothionein; TCEP, tris(2-carboxyethyl)phosphine

**Speciation resulting from ^63^Cu(I) addition to ^68^Zn_7_-MT1A, ^68^Zn_7_-MT2, and ^68^Zn_7_-MT3**





Figure S1 Speciation resulting from ^63^Cu(I) addition to ^68^Zn_7_-MT1A (A), MT2 (B), and MT3 (C) generated from corresponding ESI-mass spectral data (reported in references 27, 24, and 28). Only species reaching abundance of >5% are shown.
